# Editorial: Advanced motion control and navigation of robots in extreme environments

**DOI:** 10.3389/frobt.2024.1510013

**Published:** 2024-11-15

**Authors:** Allahyar Montazeri, Nargess Sadeghzadeh-Nokhodberiz, Khoshnam Shojaei, Kaspar Althoefer

**Affiliations:** ^1^ School of Engineering, Lancaster University, Lancaster, United Kingdom; ^2^ Department of Electrical Engineering, Qom University of Technology, Qom, Iran; ^3^ Digital Processing and Machine Vision Research Center, Najafabad Branch, Islamic Azad University, Najafabad, Iran; ^4^ School of Engineering and Materials Science, Queen Mary University of London, London, United Kingdom

**Keywords:** uncertainties, motion control, extreme environmnet, unstructured environmnet, robust and adaptive control

The use of robotics and artificial intelligence in environments which are dangerous, demanding, and dull is one of the key research areas developed recently to address the industrial need on reducing the human burden in hazardous workplace and create a safer environment for humans by taking them out of extreme environments. This may span various unstructured and cluttered environments that are difficult or dangerous for humans to access, including monitoring and sampling of deep sea, glaciers, and frozen ocean, volcanology, underground mining, space exploration, and nuclear production and decommissioning. Robotics research into challenging and cluttered environment covers Research Topic such as advanced mobility, navigation, and mapping as well as advanced motion control, manipulation, and teleoperation. Environmental characterization includes research into machine vision, remote sensing, and deep reinforcement learning.

This research need is covered in this Research Topic through nine contributions co-authored by researchers from three continents (Asia, Europe and North America). This confirms the importance of the research and development in this area across various countries and an ongoing demand for more investment.

The content of this Research Topic has been organized as follows. The first paper Mansfield and Montazeri is a review paper discusses the application of reinforcement learning (RL) in active environmental monitoring (EM) systems. The need for reliable and intelligent monitoring solutions to address environmental pollution and climate change is highlighted, with a focus on the use of RL to train agents for adaptive and robust sensing in dynamic and extreme environments. The paper proposes a framework that formulates active sensing as an RL problem, unifying various EM tasks such as coverage, patrolling, source seeking, and exploration. Despite the potential of RL for EM applications, practical implementation and research in multi-agent systems are lacking, with most work remaining in the simulation phase.

The next five papers address the navigation problems in unstructured and extreme environments. Sadeghzadeh-Nokhodbderiz et al. study the problem of attitude estimation of a quad-copter system when the quad is equipped with camera and gyroscope sensors in which cameras, usually suffer from a slow sampling rate and processing time delay compared to inertial sensors, such as gyroscopes. Toward this, a sampling importance re-sampling (SIR) particle filter (PF) is extended using a discretized attitude kinematics in Euler angles and the processing images captured by the camera using the ORB feature extraction method and the homography method in Python-OpenCV. Experimental results are provided for a DJI Tello type quadcopter to demonstrate the performance of the proposed method.

Malakouti-Khah et al. solves the problem of simultaneously localization and mapping (SLAM) for a multi-robot system in a dynamic environment. The use of several robots in large, complex, and dynamic environments can significantly improve performance on the localization and mapping task, which has attracted many researchers to this problem more recently. Toward this, a modified Fast-SLAM method is proposed by implementing SLAM in a decentralized manner by considering moving landmarks in the environment. Due to the unknown initial correspondence of the robots, a geographical approach is embedded in the proposed algorithm to align and merge their maps. Data association is also embedded in the algorithm; this is performed using the measurement predictions in the SLAM process of each robot.

The study conducted by Lim and Jo introduces WA*DH+, an improved version of WA*DH for path planning and navigation of robots in the extreme environments. WA*DH struggles to find suboptimal nodes due to its filtering method, so the study inflated the suboptimality of the initial solution. WA*DH + uses the GBFS algorithm with an infinitely bounded suboptimal solution, resulting in faster solution returns than WA*DH.

The work in Sadeghzadeh-Nokhodberiz et al., however, addresses the inter-agent collision avoidance problem for a group of quadcopters cooperate each other for a totally distributed collision-free formation tracking control using Barrier Lyapunov function (BLF). The problem is formulated in a backstepping setting where both tracking and inter-agent collision avoidance are obtained through a predefined accuracy due to the use of BLFs. Virtual control inputs are considered for the translational (*x* and *y*-axes) subsystems that are then used to generate the desired values for the roll and pitch angles for the attitude control subsystem to solve the underactuated nature of the system leading to a hierarchical controller structure for each quadcopter. Finally, the attitude controller is designed for each quadcopter locally by taking into account a predetermined error limit by another BLF. Simulation results demonstrate the performance of the proposed approach.

Nevertheless, the fifth paper on navigation published by Sands has incorporated optimality criteria in problem formulation. Optimization techniques are useful for autonomous navigation but face challenges like noisy multi-sensor technologies and computational burdens. This study aims to highlight the efficacy and limitations of common methods and proposes more, applying them to full, nonlinear, coupled equations of motion. Five different types of optimum guidance and control algorithms are presented and compared to a classical benchmark. Real-time optimization with singular switching and nonlinear transport theorem decoupling is introduced, showing superior performance in tracking errors, fuel usage, and computational burden.

The investigation by Hathaway et al. addresses the need of teleoperation in challenging environments. The use of telerobotics for semi-autonomous robotic disassembly of electric vehicle batteries is studies in this work. It compares a traditional haptic-cobot framework with identical cobots, revealing a time reduction of 22%–57%. However, this improvement is mainly due to expanded workspace and 1:1 positional mapping, and a 10%–30% reduction in first attempt success rate. The study also highlights the importance of realism in directional information for unbolting and grasping tasks.

The last paper is dealing with designing advanced motion controllers for robotics applications in front of external disturbances and uncertainties. Nguyet and Ba introduces the task-space position-tracking control of robotic manipulators using an adaptive robust Jacobian-based controller. The controller’s structure is based on the conventional Proportional-Integral-Derivative (PID) paradigm. To compensate for both internal and external disturbances in the robot dynamics, an additional neural control signal is then synthesized under a non-linear learning law. Then, a novel gain learning feature is included to automatically change the PID gains for different operating situations, providing the high robustness of such a controller. Lyapunov constraints ensure the closed-loop system’s stability. Results from extensive simulations are used to rigorously verify the suggested controller’s effectiveness.

