# Corrigendum: Editorial: Advanced motion control and navigation of robots in extreme environments

**DOI:** 10.3389/frobt.2025.1593019

**Published:** 2025-04-04

**Authors:** Allahyar Montazeri, Nargess Sadeghzadeh-Nokhodberiz, Khoshnam Shojaei, Kaspar Althoefer

**Affiliations:** ^1^ School of Engineering, Lancaster University, Lancaster, United Kingdom; ^2^ Department of Electrical Engineering, Qom University of Technology, Qom, Iran; ^3^ Digital Processing and Machine Vision Research Center, Najafabad Branch, Islamic Azad University, Najafabad, Iran; ^4^ School of Engineering and Materials Science, Queen Mary University of London, London, United Kingdom

**Keywords:** uncertainties, motion control, extreme environmnet, unstructured environmnet, robust and adaptive control

In the published article, there was an error in **Affiliation 3**. Instead of “Department of Electrical Engineering, Islamic Azad University Najafabad, Najafabad, Iran,” it should be “Digital Processing and Machine Vision Research Center, Najafabad Branch, Islamic Azad University, Najafabad, Iran.”

The authors apologize for this error and state that this does not change the scientific conclusions of the article in any way. The original article has been updated.

